# Umbilical cord as a long-term source of activatable mesenchymal stromal cells for immunomodulation

**DOI:** 10.1186/s13287-019-1376-9

**Published:** 2019-09-23

**Authors:** Anton Selich, Katharina Zimmermann, Michel Tenspolde, Oliver Dittrich-Breiholz, Constantin von Kaisenberg, Axel Schambach, Michael Rothe

**Affiliations:** 1 Institute of Experimental Hematology, Hannover Medical School, Hannover, Germany; 2 Department of Gastroenterology, Hepatology & Endocrinology, Hannover Medical School, Hannover, Germany; 3 Hannover Medical School, Central Core Unit Transcriptomics, Hannover, Germany; 4 Department of Obstetrics and Gynecology, Hannover Medical School, Hannover, Germany; 5 REBIRTH Cluster of Excellence, Hannover Medical School, Hannover, Germany; 6 Division of Hematology/Oncology, Boston Children’s Hospital, Harvard Medical School, Boston, MA USA

**Keywords:** Umbilical cord, Clonal assays, DNA barcoding, Immunosuppression, Mesenchymal stromal cells, Secretome, Transcriptome

## Abstract

**Background:**

Mesenchymal stromal cells (MSCs) are used in over 800 clinical trials mainly due to their immune inhibitory activity. Umbilical cord (UC), the second leading source of clinically used MSCs, is usually cut in small tissue pieces. Subsequent cultivation leads to a continuous outgrowth of MSC explant monolayers (MSC-EMs) for months. Currently, the first MSC-EM culture takes approximately 2 weeks to grow out, which is then expanded and applied to patients. The initiating tissue pieces are then discarded. However, when UC pieces are transferred to new culture dishes, MSC-EMs continue to grow out. In case the functional integrity of these cells is maintained, later induced cultures could also be expanded and used for cell therapy. This would drastically increase the number of available cells for each patient. To test the functionality of MSC-EMs from early and late induction time points, we compared the first cultures to those initiated after 2 months by investigating their clonality and immunomodulatory capacity.

**Methods:**

We analyzed the clonal composition of MSC-EM cultures by umbilical cord piece transduction using integrating lentiviral vectors harboring genetic barcodes assessed by high-throughput sequencing. We investigated the transcriptome of these cultures by microarrays. Finally, the secretome was analyzed by multiplexed ELISAs, in vitro assays, and in vivo in mice.

**Results:**

DNA barcode analysis showed polyclonal MSC-EMs even after months of induction cycles. A transcriptome and secretome analyses of early and late MSC cultures showed only minor changes over time. However, upon activation with TNF-α and IFN-γ, cells from both induction time points produced a multitude of immunomodulatory cytokines. Interestingly, the later induced MSC-EMs produced higher amounts of cytokines. To test whether the different cytokine levels were in a therapeutically relevant range, we used conditioned medium (CM) in an in vitro MLR and an in vivo killing assay. CM from late induced MSC-EMs was at least as immune inhibitory as CM from early induced MSC-EMs.

**Conclusion:**

Human umbilical cord maintains a microenvironment for the long-term induction of polyclonal and immune inhibitory active MSCs for months. Thus, our results would offer the possibility to drastically increase the number of therapeutically applicable MSCs for a substantial amount of patients.

**Electronic supplementary material:**

The online version of this article (10.1186/s13287-019-1376-9) contains supplementary material, which is available to authorized users.

## Background

Mesenchymal stromal cells (MSCs) are used in a variety of clinical trials for a plethora of diseases, due to therapeutically relevant characteristics such as their differentiation potential, migration to tumors, and interaction with the immune system [1–3]. MSCs can be isolated from virtually all tissues since the culture-initiating cells reside in the perivascular vicinity, but over 80% of all clinical trials use bone marrow (BM), umbilical cord (UC), and adipose tissue (AT)-derived MSCs [2, 4, 5]. UC is a very attractive source for MSCs due to the high availability, the non-invasive and ethically unproblematic isolation procedure and the higher expansion capacity compared to adult tissues [6–8]. In contrast to BM and AT, where cells are singularized, the majority of UC-MSCs are derived from approximately 100 cultivated UC pieces (UCPs) from a single UC preparation [9–14]. UC contains a niche, from which MSC explant monolayers (MSC-EMs) can grow out for several months [15, 16]. Once an MSC-EM has been initiated, the UCPs can be transferred to another dish for the next MSC culture. Despite the possibility to harvest MSC for months from a single UC preparation, clinical investigators expand and transplant only the first outgrown cells, which usually needs 1–2 weeks [9–14]. In case later initiated cultures retain their functional properties over time, even a few induction cycles would already multiply the amount of therapeutically available MSC, surpassing cell numbers that can be generated from other sources. MSC are characterized by a set of minimal criteria defined by the International Society for Cell & Gene Therapy (ISCT) [17, 18]. However, these minimal criteria do not describe any functional parameters used in clinical trials. Even though the majority of clinical trials rely on the immune modulatory capacity of MSC due to their rich secretome, there are no functional release criteria [5, 18, 19]. Here, we characterized the secretome, transcriptome, clonal development, and the immune modulatory capacity of MSC-EM after different induction time points. First, we markedly improved the transduction of UCPs by targeting the proposed stem cell niche to analyze the clonal development of initiated MSC-EMs with integrating lentiviral vectors harboring a barcode library. The most effective preparation method, the complete removal of the blood vessel (BLRV), increased the lentiviral transduction efficiency and decreased the induction time of MSC-EM. Genetic barcode vector transduction of the UCPs in combination with high-throughput sequencing of the MSC-EMs showed a highly polyclonal pattern for BLRV in contrast to the other preparation methods. To better characterize the factors secreted by MSC-EM, we used the Bio-Plex Pro™ Human Cytokine 27-plex Assay to analyze the secretome of early (initial MSC-EM, 14 days post-UCP preparation) and late (MSC-EM, 2 months post-UCP preparation) induced MSC-EMs upon activation with IFN-γ and TNF-α. IFN-γ and TNF-α partially mimic an inflammatory environment, and several groups showed higher cytokine expression by MSC upon activation [20, 21]. We observed continuously increasing production of cytokines from late induced MSC-EMs. Since the secretome of MSCs is complex and extracellular vesicles might contribute to an immunomodulatory effect, it is difficult to model this activity in vitro. Hence, we tested the effect of conditioned media a mixed lymphocyte reaction assay (MLR) and in an in vivo killing assay of allogeneic peripheral blood mononuclear cells (PBMCs) [22]. We observed a significantly higher T-cell inhibition with conditioned medium from late induced MSC-EMs, suggesting a higher immune inhibitory capability compared to early induced MSC-EMs. Our findings show a simple method for a continuous supply of genetically modified MSC by only one transduction of UCPs and most important a persistent immune inhibitory capability beyond the first induced MSC-EMs. Thus, this technology could drastically increase the number of immune modulatory MSC for a substantial amount of patients.

## Methods

### UC and MSC culture

**Fig. 1:**
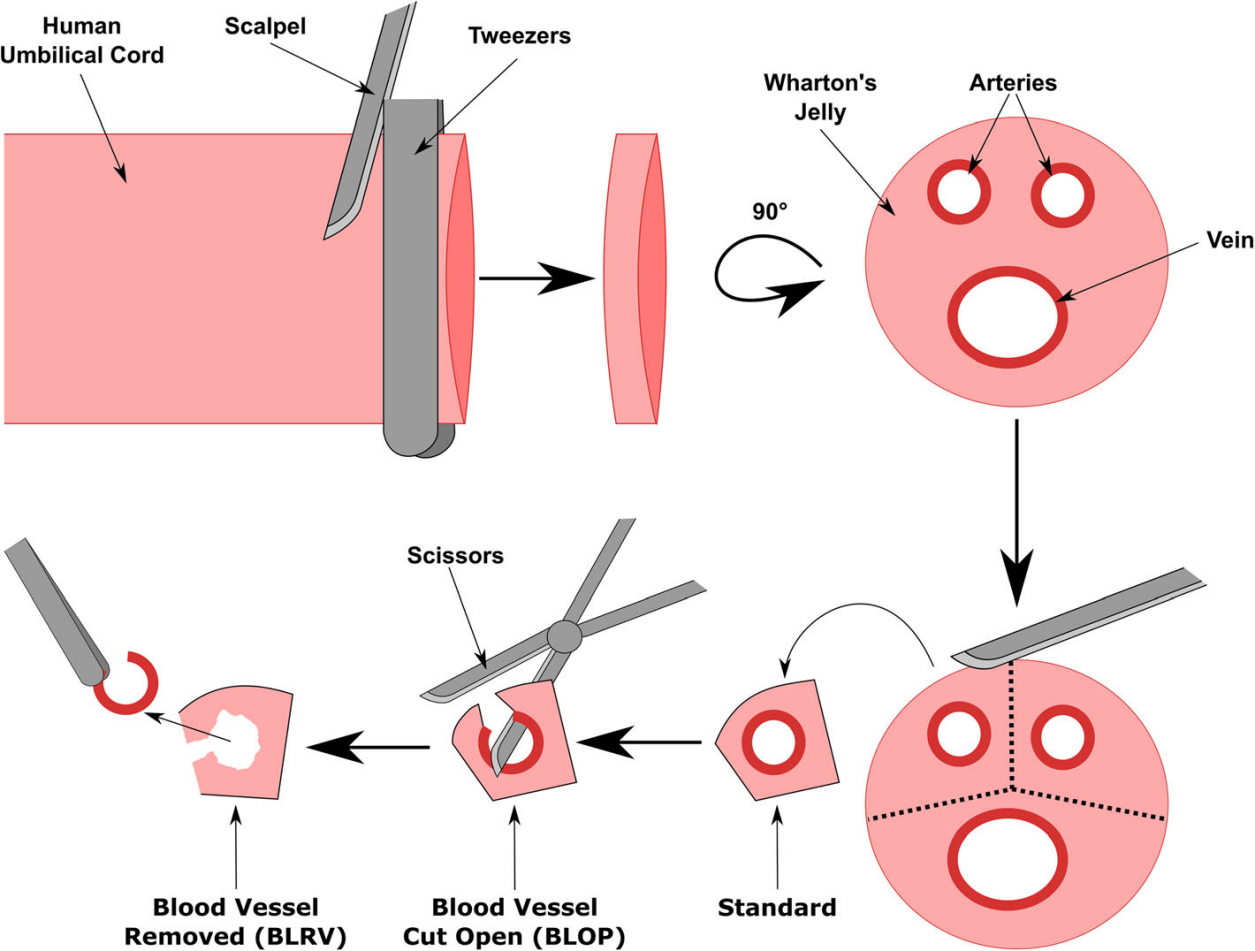
Schematic description for human umbilical cord preparation to improve the transduction of MSC-EM-initiating cells. Tweezers (4 mm) were used to generate uniform slices by transversal dissection of the umbilical cord with a scalpel. In the second step, the slices were cut between the blood vessels to ensure one blood vessel in each of three pieces (Standard). One blade of the scissors was pushed into the blood vessel lumen and closed in the orientation that the second blade cut through the surface of the umbilical cord and opened the blood vessel (BLOP). In the last step, the blood vessel was removed entirely by simply pulling out the blood vessel (BLRV)

Human UC was obtained from term deliveries (38–40 weeks) of healthy mothers after written informed consent, in accordance with the standards of the Hannover Medical School Ethics Committee and with the Helsinki Declaration of 1975, as revised in 1983. UC pieces were prepared as described in Fig. 1 and cultivated in MSC15 (MEM α, GlutaMAX™ Supplement, no nucleosides (Thermo Fisher Scientific), 15% hAB-serum (C.C.Pro GmbH), 1% penicillin/streptomycin (PAN-Biotech)). An MSC-EM was rated as induced when approximately 10^5^ cells grew in the well. To expand the cells from 12-well (Sarstedt) to 6-well plates (Sarstedt) for subsequent analysis, we transferred the UCPs with forceps to a new well of a 12-well plate to induce the next MSC-EMs. The induced MSC-EMs were detached with trypsin (PAN-Biotech) and terminated by the addition of the culture supernatant. Cells were pelleted by centrifugation (400×*g*, 5 min), resuspended in 3 mL MSC10 (reduced hAB-serum to 10%) and seeded in a 6-well plate. Since Otte and colleagues analyzed in detail the UC explant culture-derived cells, and we confirmed the MSC phenotype in our previous work, we confined only to the analysis of CD73, CD90, and CD105 (Additional file 1: Figure S1) [16, 17].

### Virus production and transduction

Third-generation lentiviral SIN vectors were produced using a four-plasmid split packaging system as described before and stored at − 80 °C until use [23, 24]. The spleen focus-forming virus (SFFV) promoter was replaced by the chromatin-opening element (CBX3) in combination with the elongation factor 1α short promoter (EFS) to reduce the risk of enhancer-mediated insertional mutagenesis [16, 25]. The vectors contain one fluorescent marker (Cerulean, mCherry, or Venus) and enable a color mixture after triple transduction to provide a first insight into clonal development by flow cytometry. Additionally, the vectors harbor a genetic barcode region composed of 15 fluorescent-specific and 16 random nucleotides, which were analyzed by high-throughput sequencing for high-resolution analysis of clonal development. Umbilical cords were cut in small pieces (Fig. 1), intensively washed with PBS, cultivated for 4 h in MSC15, washed with PBS, cultivated for an additional 20 h and washed with PBS to remove glycol precipitates as otherwise most viral particles will stick to viscid exudate. The UCPs were transduced in a final volume of 500 μL MSC10 virus mix per well (0.46 × 10^8^, 0.92 × 10^8^, or 2.12 × 10^8^ transduction units (T.U.)/mL) of a 24-well plate (Sarstedt) supplemented with 4 μg/mL protamine sulfate (Sigma-Aldrich) for 18 h. Standard cultivation medium contains non-heat inactivated serum to prevent degradation of growth factors, but transduction medium contained inactivated serum (20 min, 56 °C) to prevent the destruction of viral particles by the complement system. UCPs were transferred to a 12-well plate (Sarstedt) and cultivated in MSC15.

### Cytokine analysis

MSC-EMs from different induction time points were either cultivated in MSC10 or activated with different concentrations of IFN-γ (PeproTech) and TNF-α (PeproTech) for 4 days (+ 0, 1, 5, and 25 ng/mL). Afterwards, cells were washed with PBS, and fresh MSC10 was added for 24 h. Medium was collected and stored for later analysis at − 20 °C. Supernatants were analyzed with Bio-Plex Pro™ Human Cytokine 27-plex Assay (BioRad) according to the manufacturer’s protocol. MSC10 without cells were used as controls. Medium cytokine levels determined in controls were subtracted from cytokine levels measured in each sample to obtain the net change secreted by the cells. Since cells grew differently, we divided the net cytokine amount by the area under the exponential growth curve.

### Deep sequencing preparation

Cells were harvested by centrifugation (400×*g*, 5 min), the supernatant was removed, and pellets were stored at − 20 °C. The DNA was isolated with the QIAamp DNA Blood Mini Kit (Qiagen). The amplicon preparation was performed as described before [16]. In short, the barcodes were amplified in a nested PCR. To distinguish different samples in high-throughput sequencing, one of the inner PCR primers contained a sample specific tag. The pooled samples were sequenced in the Ion TorrentTM PGM system (Thermo Fisher Scientific).

High-throughput sequencing results were searched for the primer tags to assign the sequences to the different samples with a custom Perl 5 script (https://www.perl.org). In a second step, the sequences were screened for the barcode flanking sequences “TACCATCTAGA” and “CTCGAGACT” to remove unspecific amplicons. The last step of preprocessing was performed with a custom R (https://www.R-project.org/) script. The barcodes contain 16 random nucleotides with a potential of several billion unique barcodes. Unlikely close barcodes were assigned as the same and summed to the most frequent (clustering). The stacked bar charts were built with a custom R-script.

### Transcriptome analysis

Cells were harvested by centrifugation (400×*g*) for 5 min, resuspended in 700 μL of RNAzol B reagent (WAK-Chemie Medical) and frozen at − 80 °C. Total RNA was isolated with the Direct-Zol RNA MiniPrep Kit (Zymo Research) and on-column DNAse treatment. Microarrays utilized in this study represent a refined version of the Whole Human Genome Oligo Microarray 4x44K v2 (Design ID 026652, Agilent Technologies), called “054261On1M” (Design ID 066335) developed by the Research Core Unit Genomics (RCUG) of Hannover Medical School. Microarray design was created at Agilent’s eArray portal using a 1 × 1M design format for mRNA expression as a template. All non-control probes of design ID 026652 were printed five times within a region comprising a total of 181,560 features (170 columns × 1068 rows). Four of such regions were placed within one 1 M region giving rise to four microarray fields per slide for individual hybridization (customer-specified feature layout). Control probes required for proper Feature Extraction software operation were determined and placed automatically by eArray using recommended default settings. One hundred nanograms of total RNA was used to prepare aminoallyl uracil triphosphate (aaUTP)-modified cRNA (Amino Allyl MessageAmp™ II Kit; #AM1753; Thermo Fisher Scientific) applying one round of amplification as directed by the manufacturer, except for a twofold downscaling of all reaction volumes. Before the reverse transcription reaction, 1 μL of a 1:5000 dilution of Agilent’s One-Color spike-in Kit stock solution (#5188-5282, Agilent Technologies) was added to 100 ng of total RNA of each analyzed sample. Alexa Fluor 555 Reactive Dye (#A32756; Thermo Fisher Scientific) was used to label aaUTP-cRNA as recommended in the manual of the Amino Allyl MessageAmp™ II Kit (two-fold downscaled reaction volumes). cRNA fragmentation, hybridization, and washing steps were accomplished as recommended in the “One-Color Microarray-Based Gene Expression Analysis Protocol V5.7,” except that 700 ng of each fluorescently labeled cRNA population was used for hybridization. Slides were scanned using the Agilent Microarray Scanner G2565CA (pixel resolution 3 μm, bit depth 20). Data extraction was performed with the “Feature Extraction Software V10.7.3.1” using the extraction protocol file “GE1_107_Sep09.xml.” Microarray results were processed with the R-package limma, Rtsne, and ggplot2 [26–28]. Results describing early passages refer to the first induced MSC-EMs. Data from late MSC-EM cultures were obtained after the sixth (*n* = 1) or seventh (*n* = 8) initiating cycle. Data from activated cultures were obtained from two late MSC-EMs. Differential expression was calculated with the commands lmFit and eBayes with the contrasts “Activated-Late,” “Activated-Early,” and “Early-Late” from the limma package. Lists contain only genes with a Bonferroni-Holm corrected *p* value below 0.05. Lists of differentially expressed genes were analyzed with Ingenuity Pathway Analysis [29], with an absolute analysis threshold of a log2 expression difference of 1. The scoring method for the Canonical Pathway (CP) analysis used Benjamini-Hochberg (B-H)-corrected *p* values. CP, Diseases, and Function (DF), as well as Upstream Regulator (UR) tables, were exported with all available columns. UR tables were filtered for category genes, RNAs, and proteins with an absolute *z*-score ≥ 2 and sorted for the product of the negative logarithmic BH-corrected *p* value and the activation *z*-score. CP tables were sorted for the product of the logarithmic BH-corrected *p* value and the ratio of matching molecules in the respective pathway.

### Mixed lymphocyte reaction assay

1 × 10^5^ Ficoll-purified human PBMC from two HLA-mismatched donors were mixed, cultivated in 200 μL in media based on MSC10 (+20 mM HEPES (PAN Biotech), + 50 μM mercaptoethanol (Sigma-Aldrich)) in 96-well round bottom suspension plates (Sarstedt). The negative control was only medium without additional 400 IU/mL IL2 (Novartis). Conditioned media from early and late induced MSC-EM were harvested as described before for the cytokine analysis (activation: 25 ng/mL IFN-γ, 1 ng/mL TNF-α). PBMCs from the mismatched donors were cultivated separately in the respective media for 24 h and subsequently mixed in a 1:1 ratio. 90 % of the medium was replaced by fresh respective media after 3 days. Cells were analyzed after an additional 4 days by flow cytometry. Three MSC donors were tested with each conditioned medium and each donor was evaluated as a technical triplicate. One data point was removed from the analysis due to a positive Grubbs outlier test. Statistical analysis was performed with GraphPad Prism 6 (GraphPad Software, Inc.) using One-way ANOVA, Tukey- Post-Hoc tests.

### In vivo killing assay

Animal experiments were approved by the supervising animal research review board at Hannover Medical School and the Lower Saxony State Office for Consumer Protection and Food Safety. The in vivo killing assay was performed as previously published [22]. In short, mice (non-obese diabetic (NOD)-RAG1^null^IL2c^null^) were intraperitoneally reconstituted with 7.5 × 10^6^ Ficoll-purified human PBMCs. The reconstitution was controlled 14 days later by flow cytometric analysis of the peripheral blood. Syngeneic (with respect to the reconstituted human immune system in the mice) and allogeneic PBMCs were cultivated for 18 h in a basic medium (MSC10) or conditioned media from activated early or late induced MSC-EMs. Conditioning was performed as described in “Cytokine analysis” with IFN-γ (25 ng/mL) and TNF-α (1 ng/mL). Syngeneic cells were marked with eFlour670 (eBioscience) and allogeneic with CFSE (Invitrogen) and mixed according to the respective conditioned medium in a 1:1 ratio (5 × 10^5^ cells), prior to intravenous injection into reconstituted mice (200 μL/mouse). The mice were sacrificed 5 days later, and cells from the peripheral blood and spleen were analyzed by flow cytometry. Cells were stained with Zombie yellow (BioLegend) and hCD3-BV421 antibody (BioLegend). Percentages of allogeneic cells within living (Zombie yellow negative) injected (APC+ or CFSE+) hCD3+ cells were plotted and statistically analyzed (two-way ANOVA, Tukey post hoc) with GraphPad Prism 6 (GraphPad Software, Inc.).

### Statistical analyses

**Fig. 2:**
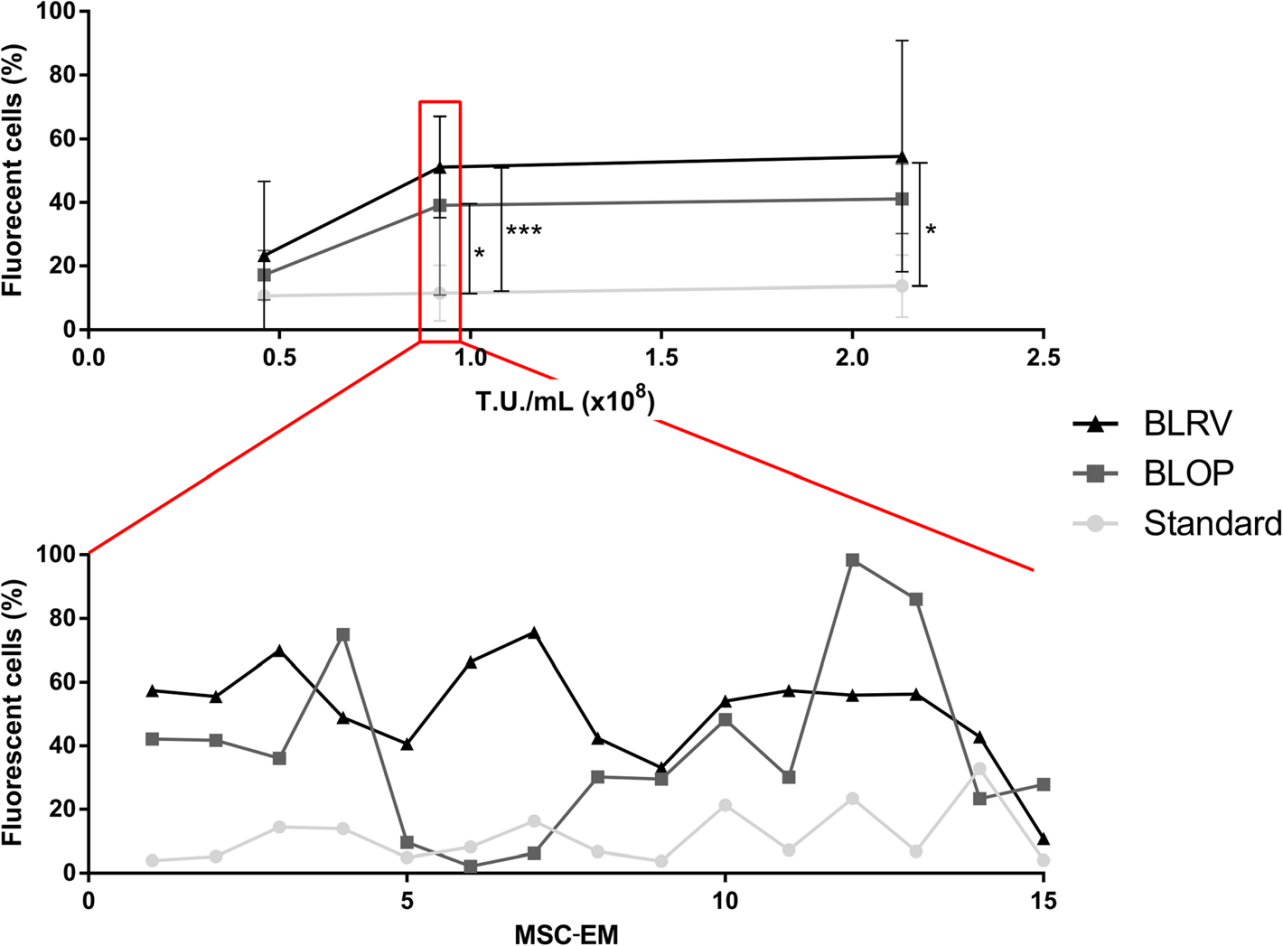
Removal of the blood vessel markedly increased the transduction of MSC-EM-initiating cells in the UCPs. To compare the transduction efficiency of differently prepared UCPs, each donor UC was prepared in all methods, and different donors were used for different concentrations of viral vectors coding for fluorescent reporter proteins. The outgrown MSC-EMs were analyzed by flow cytometry. In the upper graph, the *x*-axis shows the different vector concentration, the *y*-axis the percentage of fluorescent-protein-positive cells in induced MSC-EMs. In the lower graph, the variation of gene marking among MSC-EMs from the same UCPs is shown. Depicted are the means and the standard deviations. *n* > 7. Statistical tests were performed with Kruskal-Wallis and Dunn’s multiple comparison tests. Only significant differences are indicated. * = *p* < 0.05, *** = *p* < 0.001, T.U. = transduction units, MSC-EM = mesenchymal stromal cell explant monolayer, Std = standard preparation, BLOP = blood vessel cut open, BLRV = blood vessel completely removed

**Fig. 3:**
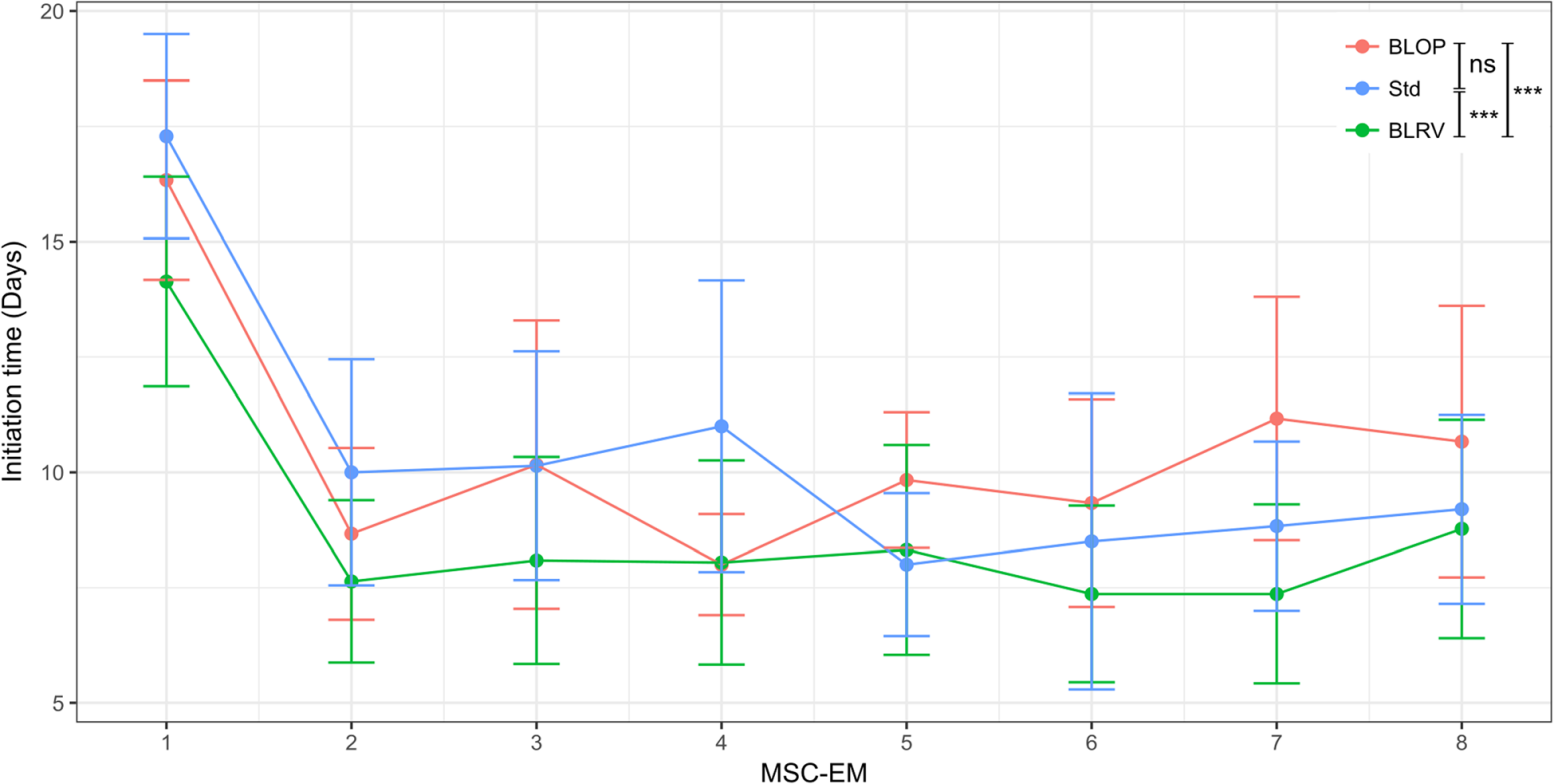
Removal of the blood vessel reduced the initiation time of MSC-EMs. The time between consecutive MSC-EMs (induction time) was observed for approximately 2 months for the different preparation methods. A culture was rated as induced when the cell number reached approximately 10^5^ cells in a well. Depicted are the means and the standard deviations. As the first initiation of an MSC-EM usually takes longer than all consecutive MSC-EMs, we excluded the first time point from the statistical analysis. A Kruskal-Wallis test revealed significant differences among the preparation methods (*p* = 3.2e−07). A subsequent pairwise Wilcoxon-Rank-Sum test with Bonferroni-Holm correction showed significant differences for BLRV vs BLOP (*p* = 1.4e−05), BLRV vs Std (*p* = 8.5e−05), and no significant differences between Std vs BLOP (*p* = 0.88). *n* = 5–22 (at least 4 donors for each preparation method). Std = standard preparation, BLOP = blood vessel cut open, BLRV = blood vessel completely removed

The statistical significance for Fig. 2 was calculated with Kruskal-Wallis and subsequent Dunn’s multiple comparisons (GraphPad Prism 6). For Fig. 3, significant differences were determined by a Kruskal-Wallis test and a pairwise Wilcoxon rank sum test with Bonferroni-Holm correction (https://www.R-project.org/). For the statistical analysis of the secretome, we applied a Wilcoxon signed-rank test (https://www.R-project.org/). The pairing parameter was a combination of produced cytokine and amount of cytokines used to activate the cells (e.g., IL-6 concentration of early MSC-EMs after activation with 1 ng/mL TNF-α and IFN-γ vs. IL-6 concentration of late early MSC-EMs after activation with 1 ng/mL TNF-α and IFN-γ).

## Results

### Removing blood vessels from umbilical cord increases accessibility to MSC-initiating cells

Since clonal dominance after extended culture time raises concerns of transformation, we aimed to analyze the clonal composition of consecutive MSC-EMs. In a previous work, we showed the transduction of whole UCPs and the outgrowth of genetically modified MSCs [16]. However, cells on the surface of the UCPs were preferentially transduced and limited the number of genetically modified cells in initiated MSC-EMs. Our standard preparation method of UCPs (further referred to as Std) preserves all parts of the UC (blood vessels and Wharton’s jelly), including the MSC-initiating cell niche (Fig. 1). We hypothesized that transduction of cells in the vicinity of blood vessels, the proposed MSC niche, would markedly increase the transduction efficiency. To increase the accessibility of this niche, we either cut the blood vessels to open them (BLOP) or completely removed them (BLRV). Following the different UCP preparation methods, we transduced the tissue pieces by simply cultivating the UCPs in different vector concentrations for 18 h and analyzed the induced MSC-EMs by flow cytometry (Fig. 2). While both optimized preparation methods increased the proportion of transgene-positive cells in induced MSC-EMs, removal of the blood vessels led to the most efficient transduction of MSC-initiating cells (Fig. 2). All UCPs were able to induce MSC cultures over the entire experimental period of at least eight initiating cycles.

### Removal of blood vessels does not destroy the MSC-initiating cell niche

**Fig. 4:**
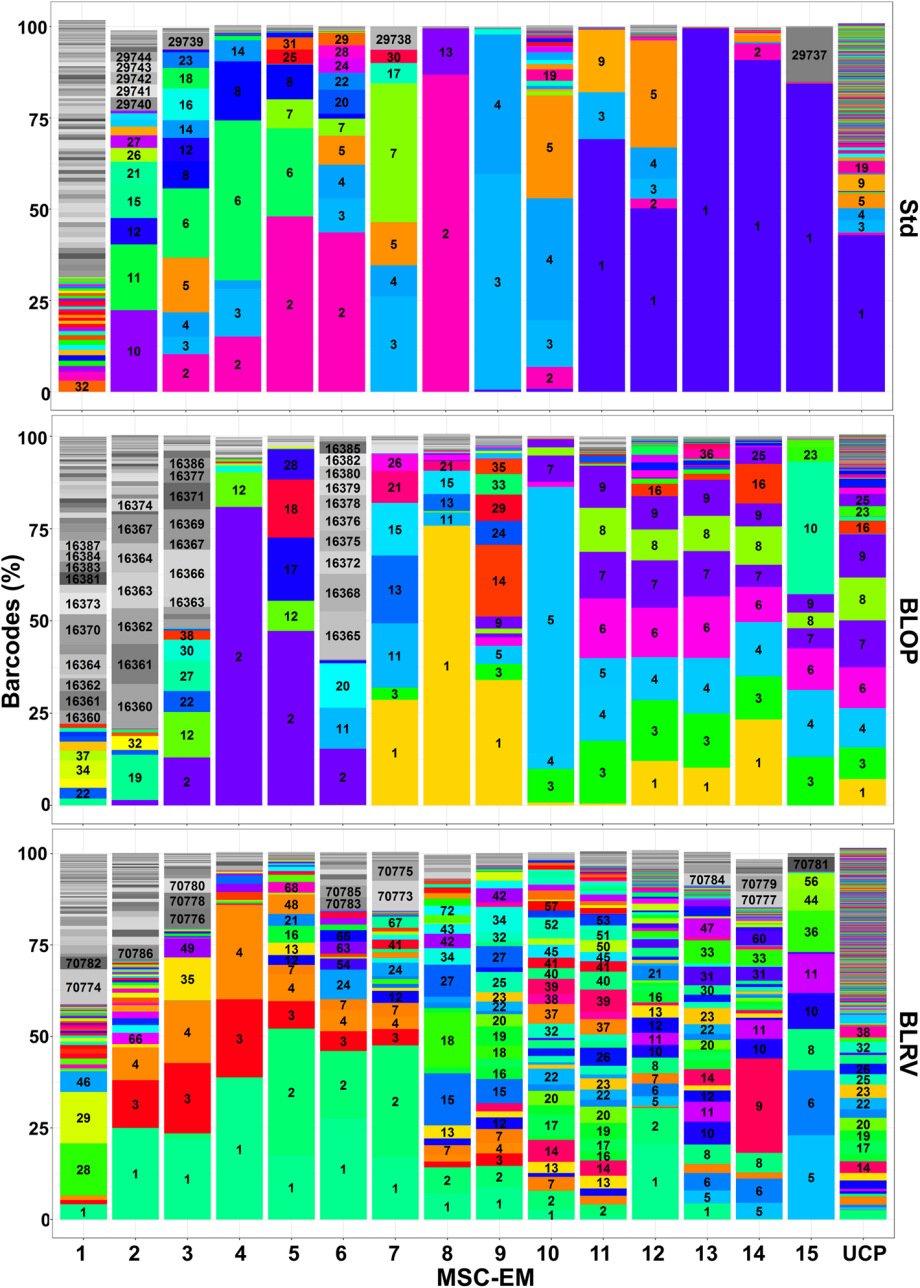
Clonal analysis of MSC-EMs and UCPs using lentiviral barcoding and high-throughput sequencing. Differently prepared UCPs were transduced with lentiviral barcode vectors 1 day after preparation. The barcodes of the first 15 induced MSC-EMs and the corresponding UCPs were analyzed by high-throughput sequencing. The areas in the stacked bar chart represent the abundance of barcodes. Unique numbers label different reoccurring barcodes for each condition. The areas in different shades of gray represent barcodes, which were not present in the UCPs at the end of the analysis. UCP = umbilical cord piece, BLOP = blood vessel cut open, BLRV = blood vessel completely removed, MSC-EM = mesenchymal stromal cell explant monolayer

The advantage of UCP explant cultures over other MSC sources, e.g., bone marrow, is the possibility for continuous initiation of MSC cultures over several months. To investigate potential influences of the different UCP preparation methods, we compared the initiation time of MSC-EMs among the three methods applied here (Fig. 3). BLRV induction intervals were significantly shorter compared to Std or BLOP and, as a consequence, generated more cells within the same time period. Some of the UCPs were observed over 120 days and initiated 15 MSC-EMs. UCPs were marked with genetic barcode vectors prior to initiation as described for flow cytometric analysis, to follow the clonal dynamics by high-throughput sequencing. We isolated the DNA from the consecutive induced MSC-EMs and the UCPs after induction of the 15th batch of MSC-EMs. In Fig. 4, the stacked bar charts show the abundance of different barcodes (*y*-axis) in consecutive MSC-EMs as well as the inducing UCPs (*x*-axis). All barcodes, which were found in MSC-EMs and the respective UCPs, are shown with a distinct color and barcode number. All barcodes observed in MSC-EMs, but which were not found in the UCPs, are shown in different shades of gray. The early induced MSC-EMs showed the highest variability of barcodes, but most of them were not found in the initiating UCPs. In contrast, barcodes from MSCs of the second or third initiating cycle were also found in the UCPs at later times. The highest variability in the UCPs and the MSC-EMs was observed for BLRV. Additionally, the last 3 to 4 MSC-EMs induced by Std or BLOP were mainly composed of the same clones. In summary, Std and BLOP showed less variability in the UCPs and clonal dominance in the late induced MSC-EMs.

### Later induced MSC-EMs produce more cytokines upon activation by IFN-γ and TNF-α

**Fig. 5:**
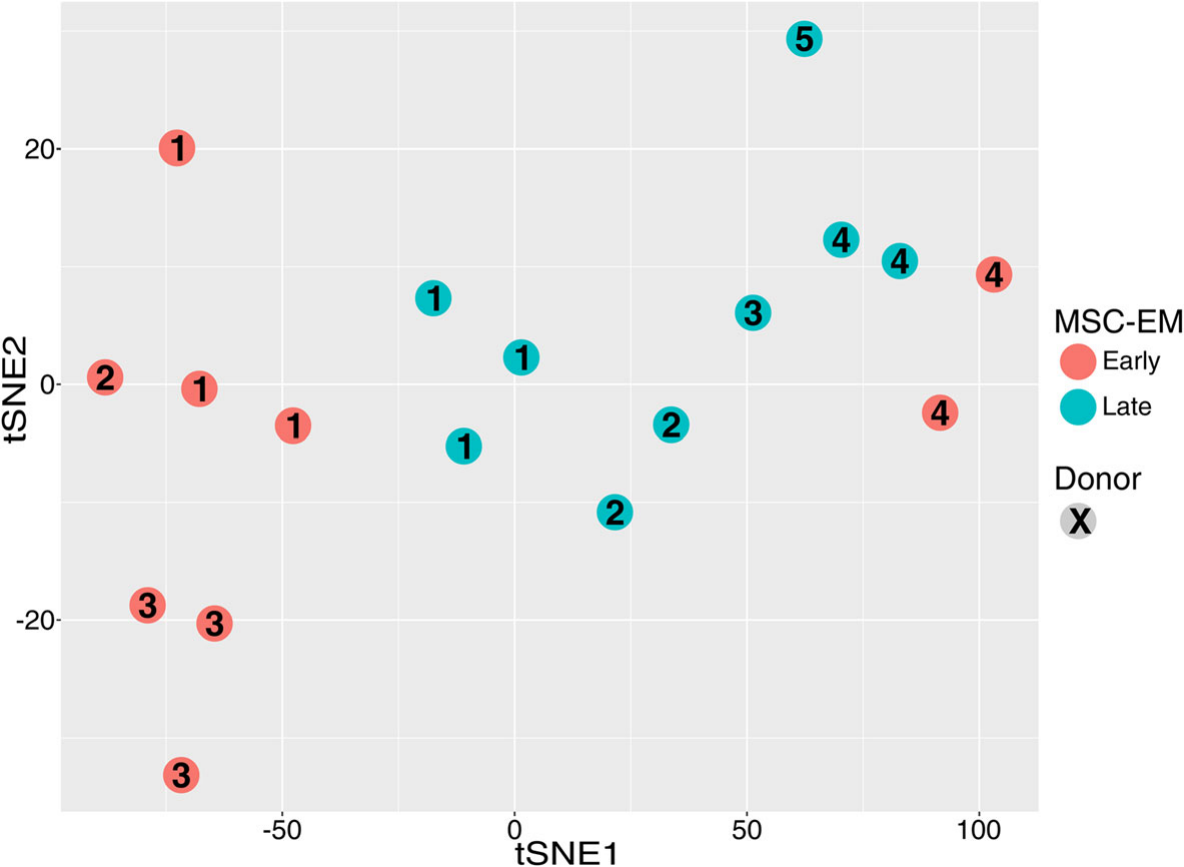
Transcriptome analysis of the different induction time points. Different induction time points were analyzed by microarray analysis. The transcriptional proximity of the samples is visualized by tSNE dimensionality reduction. All of the late (6 and 7) and most of the early time point samples (1) build separate groups. Only the first MSC from donor 4 was transcriptionally closer to late samples than to the first MSC-EMs from other donors

Since the results shown above demonstrate that removal of the blood vessels decreased the induction time and consequently provides more polyclonal MSC in the same period of time, all following experiments were done using cells derived from the BLRV method. To access the overall development of the different time points, we analyzed the transcriptome of early (2 weeks post-UCP preparation) and late (2 months after UCP preparation) induced MSC-EMs by microarray analysis (Fig. 5). The tSNE dimension reduction demonstrated slight clustering of groups by induction time. However, the overall difference in gene expression was low. The ten most deregulated genes showed an absolute mean expression difference of 12.5-fold (Additional file 2: Table S1).

**Fig. 6:**
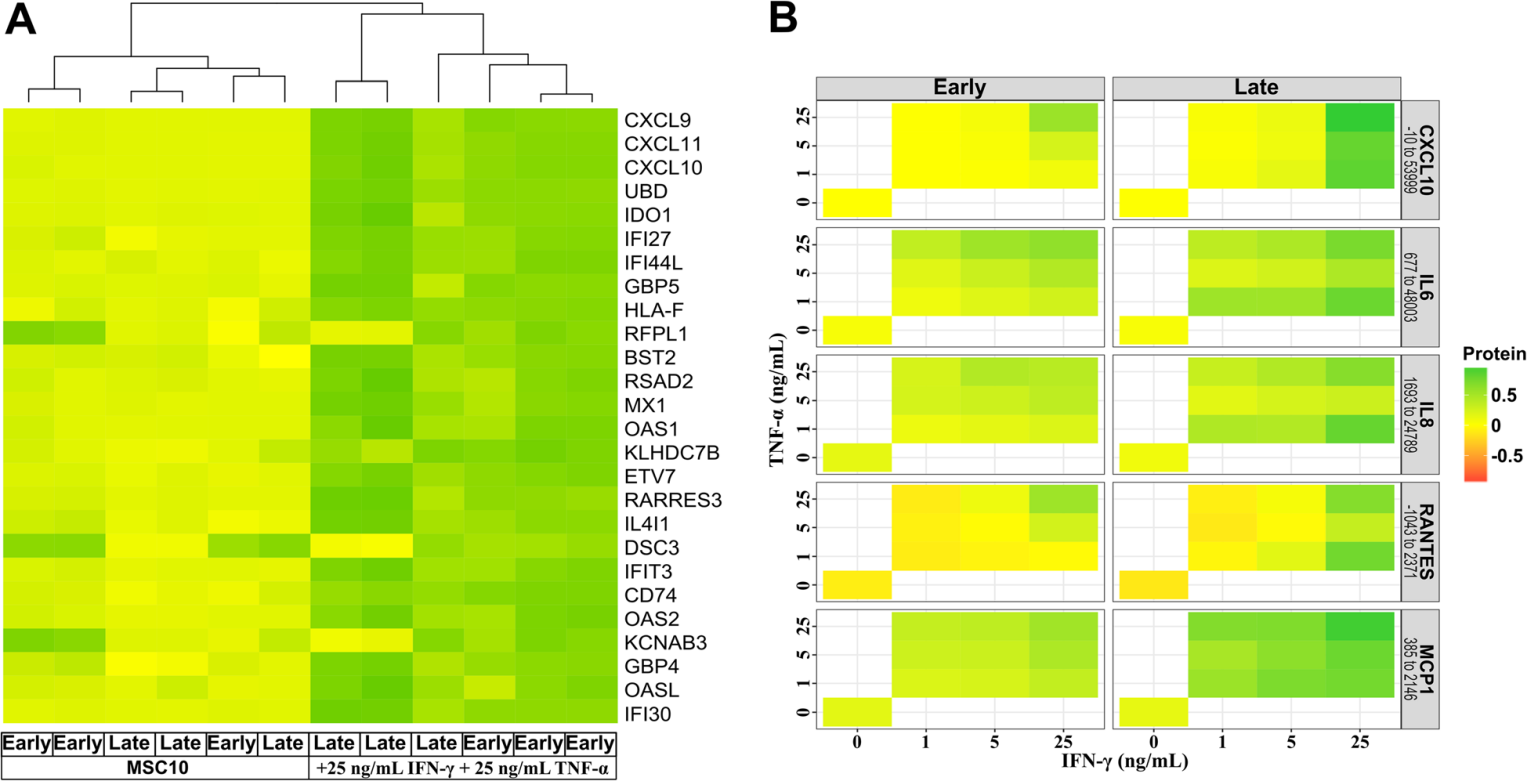
Transcriptome and secretome analyses of early and late induced MSC-EMs after activation. Early and late induced MSC-EMs were activated with IFN-γ and TNF-α for 4 days. **a** Heat map of the transcriptome analysis. **b** For conditioning, cells were washed with PBS and cultured in MSC10 for 24 h. The media were analyzed with the Bio-Plex Pro™ Human Cytokine 27-plex Assay and the 5 most deregulated cytokines are shown. Cytokines and the concentration range (pg/mL) detected are given in the column headers on the right of the graphs. A paired Wilcoxon signed-rank test was performed and confirmed significantly more produced cytokines by later induced cultures (*p* < 9.8e−06)

As a substantial amount of MSC studies investigate inflammatory diseases, we partially mimic an inflammatory environment by the addition of IFN-γ and TNF-α (+ 25 ng/mL, both) [19–21]. In contrast to the low expression differences between naïve early and late induced MSC, the addition of the cytokines drastically changed the transcriptome (Fig. 6a). The ten most deregulated genes (CXCL9, UBD, CXCL11, CXCL10, IDO1, IFI44L, IFI27, GBP5, BST2, HLA-F) showed a mean expression change of 540.8-fold compared to naïve cells (Additional file 3: Table S2). Ingenuity Pathway Analysis (IPA) identified IFN-γ and TNF-α as the major upstream regulators (Additional file 4: Table S3). Cytokine activation also led to upregulation of MHC class I/II molecules (Additional file 5: Table S4), and IPA demonstrated activation of the antigen presentation pathway (Additional file 6: Table S5). The diseases and functions analysis of IPA showed an increase in the activation of leukocytes, blood cells, and attraction and maturation of cells, with a higher activation *z*-score for later cultures (Additional file 7: Table S6).

To verify the similar reaction of early and late induced MSC-EMs upon activation with IFN-γ and TNF-α, cell culture supernatants were analyzed with the Bio-Plex Pro™ Human Cytokine 27-plex Assay (Fig. 6b). The five cytokines with the highest concentrations (CXCL10, IL-6, IL-8, RANTES, and MCP-1) are shown, and the complete set of cytokines analyzed can be found in the supplementary materials (Additional file 8: Table S7.). MSC cultures produced more cytokines after stimulation with higher concentrations of IFN-γ and TNF-α. Interestingly, late induced MSC-EMs responded better to activation signals and produced higher amounts of cytokines than early induced MSC-EMs.

### Conditioned medium from late induced MSC-EMs exert a higher immune inhibitory potential than early induced MSC-EMs in vivo

**Fig. 7:**
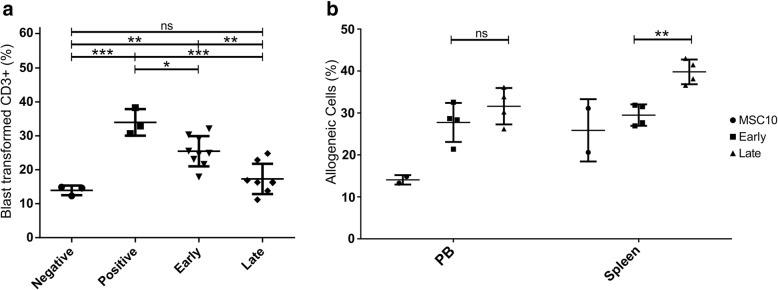
Immune inhibition of T-cells by early and late induced MSC-EMs. Early and late induced MSC-EMs were activated with IFN-γ (25 ng/mL) and TNF-α (1 ng/mL) for 4 days, washed with PBS, and used for conditioning of basic medium (MSC10) for 24 h. PBMC from HLA-mismatched donors were separately cultivated in the different media for 24 h. (**a**) For the mixed lymphocyte reaction assay, PBMC were mixed in a ratio of 1:1 and cultivated for 7 additional days. MSC10 was used as a negative control and the positive control contains IL2 (400 IU/mL). IL2 was also added to the conditioned media to activate T-cells. Conditioned medium of early and late induced MSC-EM inhibited blast transformation of T-cells. (**b**) For the in vivo killing assay, PBMC were stained with different colors, mixed medium wise, and injected in a 1:1 ratio in the respective media into mice previously reconstituted with a human immune system. Five days later, mice were sacrificed and analyzed by flow cytometry. Depicted are the percentages of allogeneic cells among all stained hCD3+ cells. Each point represents individual mice. Shown are the group means and standard deviation. Statistical tests were performed with ANOVA and *p* values calculated with Tukey’s multiple comparison. Only significant differences are indicated. ** = *p* < 0.01, *** = *p* < 0.001. PB = peripheral blood

Since we observed a higher response of late induced MSC-EMs with lower activation signals, we wanted to analyze the impact on immune regulation. Additionally, cells from long-term cultures raise the concern of genetic instability and transformation in cell therapy. As most of the beneficial effects after MSC application are presumably the result of the produced factors and extracellular vesicles, we explored the possibility to administer MSC-EM cell culture supernatants and thus avoid the transplantation of cells. We assessed the immune inhibitory effect in an MLR assay (Fig. 7a). Both conditioned media significantly inhibited the blast transformation of CD3+ cells, but only medium from late induced MSC reached levels of the negative control. Additionally, MSC-EM cell supernatants were tested in an in vivo killing assay [22], in which a mix of syngeneic and allogeneic PBMCs (1:1) was injected into humanized mice. In case of a positive immune response, syngeneic cells endure while allogeneic PBMCs are mostly eliminated by the reconstituted human immune system. PBMCs were cultivated overnight in fresh or conditioned medium, marked as allogeneic and syngeneic cells eFlour670 or CFSE and injected a 1:1 mix of allogeneic- eFlour670 and syngeneic-CFSE cells together with 200 μL of the respective culture media (either fresh or conditioned medium from activated MSC (+ 25 ng/mL IFN-γ and + 1 ng/mL TNF-α)). Mice were sacrificed after 5 days, and the proportion of allogeneic CD3-positive cells within all marked CD3 cells in the peripheral blood and spleen was determined by flow cytometry. A higher number of allogeneic cells was found in the peripheral blood of mice treated with conditioned medium as compared to fresh medium (Fig. 7b), which indicates that conditioned medium from both early and late induced MSC-EMs exhibits an immune inhibitory effect. In the spleen, a significantly higher fraction of allogeneic cells was detected in mice injected with supernatants from late induced MSC-EMs as compared to supernatants from early induced cultures.

## Discussion

MSC have gained attention as cell therapeutics mostly due to their interaction with the immune system [1–3]. UC is the second most often used source, and the majority of those trials use the explant culture method to harvest MSCs [9–14]. MSC-EMs grow out from UC for months, but only the first MSC culture is expanded and applied to patients. Here, we show that 2 months post UC preparation-induced MSC-EMs are as capable to inhibit the immune reaction as the first induced MSC-EM. Our results raise the possibility to harvest immune inhibitory MSC for months and massively increase the number of therapeutical cells for future clinical trials.

In a previous study, we used fluorescent marker genes (RGB marking) and DNA barcodes to investigate the clonal complexity of MSC culture-initiating cells of whole UCPs [16]. Transduction of UCPs was hampered by the mainly superficial labeling of tissue pieces, leading to limited amounts of transgene-positive MSCs. Since the perivascular environment is the proposed niche of MSCs, we aimed to overcome the physical barrier for lentiviral particles and to transduce cells in the vicinity of blood vessels in the UCPs. The percentage of gene-modified MSCs was significantly increased by opening or completely removing the blood vessels prior to transduction (Fig. 2). Removal of the blood vessels did not affect the ability to initiate MSC explant monolayers but resulted in up to a fivefold increase in transgene-positive cells. Interestingly, the removal of the blood vessel significantly decreased the initiation time of new MSC cultures compared to the standard method of simply culturing small intact UCPs (Fig. 3). Even though the primary purpose for lentiviral gene transfer was to study the clonal diversity and development of MSC, this preparation method could potentially be used to obtain large numbers of genetically improved MSC that express functional transgenes, e.g., immune modulators. Interleukin 10 (IL-10) is an important immunomodulatory cytokine, which was not expressed by MSCs in our study [30–32]. Lentiviral vectors could be used to equip MSC with IL-10 to further bolster their immune inhibitory properties. MSCs could also be modified to express proteins that increase cell migration to targeted tissues as described for CXCR4 overexpression to treat acute lung injury [33]. However, a careful evaluation of promoters and transgenes has to be performed to maintain the continuous outgrowth of MSCs.

To use UCPs as a constant MSC source for cell therapy, it is imperative to know whether early induced cultures are similar to those induced after prolonged culture time. Analysis of the clonal repertoire after different initiating time points following transduction of UCPs with lentiviral vectors and analyzed the overlap of barcodes between the tissue pieces and the initiated cultures. We observed the highest variability in barcodes after the first induction of MSCs (Fig. 4). However, most barcodes were only detected in the MSC culture and not in the UCPs. Already after three to four initiation cycles, MSC cultures were composed of clones, which were also detectable in the UCPs. This result suggests a hierarchical MSC system with long-term culture initiating MSC and rather short-lived progeny. This hypothesis is in line with published data that suggests the existence of MSC subpopulations with higher stem cell potential (CD146+ cells) [34]. Comparison of cultures derived from different preparation methods revealed that late induced cultures of Std and BLOP were composed of mainly the same clones, whereas BLRV showed higher clonal diversity. Removal of the blood vessel might have provided direct access to the long-term culture initiating MSCs. Removal of the physical blood vessel barrier might not only explain the higher polyclonality of the gene-modified cells, but also the reduction in initiation intervals. In an earlier study, we followed the clonal development of MSC cultured in different expansion media [35]. Interestingly, TGF-β1 supplemented medium imposed a strong selection pressure on MSC, with a marked reduction in MSC clonality. However, medium harvested from TGF-β1 selected cultures improved human hematopoietic stem cell engraftment in mice. Hence, it is difficult to say whether the most useful MSC will be derived from a well-defined oligoclonal culture with desired therapeutic features or a polyclonal culture with many different clones. Importantly, if the starting culture is oligoclonal and not further characterized, culture to culture variation with lack of therapeutically active clones in some cultures may occur leading to increased therapeutic variability between different or even the same donor. Consequently, more polyclonal initiating cultures might decrease the variability in the therapeutic outcomes but could be inferior to oligoclonal cultures consisting only of clones with desired features.

Otte and colleagues tested the UCP explant culture system for the minimal criteria of the International Society for Cell & Gene Therapy (ISCT), and describe the plastic adherence, expression of CD73, CD90, and CD105, and absence of hematopoietic markers for MSCs. In contrast to our study, they did not observe major differences between early and late induced MSC-EMs [17, 18]. The secretion of immunomodulatory factors is not a part of ISCT criteria, even though the production of cytokines and growth factors are the primary mode of action when MSCs are used in cell therapy [1, 2]. MSCs migrate to inflammatory tissues and are then exposed to inflammatory signals like TNF-α and IFN-γ [36]. Secretome analysis of early and late induced MSC-EMs after activation with different concentrations of IFN-γ and TNF-α demonstrated that the amount of cytokines produced by early and late induced MSC-EMs correlated with the concentration of the activating agents and that late induced cultures produced significantly higher levels of the cytokines (Fig. 6b). In-depth transcriptome analyses revealed low differences between early and late induced MSC-EM cultures (Fig. 5). Analysis of differentially expressed genes between activated and naïve MSCs confirmed the upregulation of various immunomodulatory genes, as described by others [20, 21, 37]. A recent MSC transcriptome analysis by Kim and colleagues found CXCL9, CXCL10, CCL8, and IDO1 as the top upregulated genes after IFN-γ stimulation [38]. From the 100 most deregulated genes, 78% overlapped with that of Kim’s study regarding up- or downregulation. Importantly, the top 5 deregulated genes in our dataset were CXCL9, CXCL11, UBD, IDO1, and CXCL10. Interestingly, CXCL9, CXCL10, and CXCL11 bind to CXCR3, attract mostly T cells, and are actually associated with an increased immune response [39–41]. Little is known about the function of Ubiquitin D in MSCs, but studies in other cell types suggested a supportive function for the antigen presentation on MHC via increased degradation of proteins, which would be in line with our observation of upregulated MHC proteins [42]. IDO, on the other hand, is a well-known protein that mediates immune inhibition by MSC [37]. To gain more insight into the mechanistic differences of gene expression changes after activation of MSC cultures with INF-y and TNF-α, we used IPA to identify expression networks and potential upstream regulators. IPA readily identified IFN-γ and TNF-α as upstream regulators likely to be causative for the observed expression differences. Pathways involved in antigen presentation, inflammation signaling, and communication between immune cells were predicted to be activated in IFN-γ- and TNF-α-stimulated MSCs. Our gene expression results imply minor differences in the gene expression of naïve early and late cultures and a preserved ability to react to activating cytokine stimulation. Donor variability has been described for many MSC applications [43, 44]. We also observed this in our study as transcriptome analyses demonstrated that early cultures of MSC-EMs from donor 4 were distinctly different from early MSC-EMs of the other donors investigated here.

To assess whether the observed differences in the secretome of early and late induced MSC cultures have functional consequences, we analyzed conditioned media of MSC cultures in an in vitro MLR and an in vivo killing assay [22]. Syngeneic and allogeneic PBMCs were cultivated in basic (MSC10) or conditioned media from either early or late induced activated MSC cultures. After overnight incubation, PBMCs were either mixed and cultivated in a 96-well plate or injected in humanized mice. Interestingly, we observed significantly less blast transformed CD3+ cells in vitro and significantly more allogeneic cells in the peripheral blood and spleen of mice where PBMCs were cultivated in MSC-conditioned media compared to fresh medium. In the spleen, we detected significantly more allogeneic cells in the group of late induced cultures compared to the other groups. In this model, the spleen harbors more effector T cells compared to circulating cells in peripheral blood, making the results more relevant for comparison of the immune inhibitory effect. As we did not filter our conditioned media prior to incubation with PBMCs or subsequent transplantation, the observed effects might not only be a consequence of the cytokines produced but may also be due to MSC-derived exosomes in the culture.

Many groups showed immune inhibitory effects of MSCs exposed to high concentrations of IFN-γ and TNF-α [20, 21]. We demonstrated an increased immunomodulatory effect of umbilical cord-derived MSCs, which is due to alteration of the secretome over time. The present work also revealed clonal restriction from early to late MSC-EMs, which may indicate a hierarchical system of long-lived progenitors and short-lived progeny. In future studies, single-cell transcriptomics could be used to investigate whether certain clones exhibited a higher immune modulatory capacity than others as suggested by Martinez-Peinado and colleagues and whether these can be actively enriched over time in mass culture [45].

## Conclusion

Here, we showed a simple method to markedly increase the transduction efficiency of UCPs to analyze the clonal development of MSC cultures and as a prospect to obtain a high number of genetically improved MSC for clinical applications. Most importantly, MSC-EMs maintained their immune inhibitory capability beyond the first induced MSC-EM culture, which is usually employed in cell therapies. Since UC is already the second leading source for MSC in hundreds of clinical trials, our findings could markedly increase the number of therapeutical MSCs for a large number of future patients.

## Additional files

Additional file 1: **Figure S1.** Antibody staining of early and late induced MSC-EMs. Early and late induced MSC-EMs were stained with antibodies for CD73, CD90, and CD105 to confirm the mesenchymal stromal cell identity of the cultures. (TIF 537 kb)

Additional file 2: **Table S1.** Significantly deregulated genes between early and late induced MSC-EMs. Contains a list of significantly deregulated genes between early and late induced MSC-EMs. *P* values were adjusted with Bonferroni-Holm method and only genes with a *p* value below 0.05 are listed. logFC = Mean of early minus mean of late induced expression. (XLSX 315 kb)

Additional file 3: **Table S2**. Transcriptome analysis of significantly deregulated genes between naïve and activated MSC-EM. Contains a list of significantly deregulated genes between naïve and activated MSC-EM. *P* values were adjusted with Bonferroni-Holm method and only genes with a *p* value below 0.05 are listed. logFC = Mean of activated minus mean of naïve MSC-EM expression. (XLSX 274 kb)

Additional file 4: **Table S3.** Contains a list of upstream regulators from Ingenuity Pathway Analysis (IPA) of the significantly deregulated genes from Additional file 3: Table S2. (XLSX 23 kb)

Additional file 5: **Table S4.** Contains a list of only significantly deregulated genes regarding MHC molecules from the IPA analysis. (XLSX 8 kb)

Additional file 6: **Table S5.** Contains a list of canonical pathways from an IPA analysis of the significantly deregulated genes from Additional file 3: **Table S2.** (XLSX 32 kb)

Additional file 7: **Table S6.** Contains a list of diseases and functions from an IPA analysis regarding significantly deregulated genes from Additional file 3: **Table S2.** (IPA). (XLSX 15 kb)

Additional file 8: Annotation and raw data from the secretome analysis. Contains the annotation of individual wells. Date: sample collection date. Medium: “I” = IFN-γ, “T” = TNF-α, 1 = 1 ng/mL, 5 = 5 ng/mL, 25 = 25 ng/mL. Count.B: Cells counted before seeding in Neubauer chamber. Squares.B: Number of squares counted before seeding. Volume.B: Solution volume of the cells for counting before seeding. Days: Conditioning time of the medium. Count.A: Cells counted after conditioning in Neubauer chamber. Squares.A: Number of squares counted after conditioning. Volume.A: Solution volume of the cells for counting after conditioning. Medium: Volume of the conditioned medium. Contains the raw data. All values are in pg/mL. (XLSX 21 kb)

## Data Availability

The raw data required to reproduce these findings are available to download from Gene Expression Omnibus (GSE117901). The processed data required to reproduce these findings are available to download from Mendeley Data [Link available prior to publication].

## Abbreviations

aaUTP: Aminoallyl uracil triphosphate
AT: Adipose tissue
B-H: Benjamini-Hochberg
BLOP: Blood vessel cut open
BLRV: Blood vessel removed
BM: Bone marrow
BST2: Bone Marrow Stromal Cell Antigen 2
CBX3: Chromatin-opening element
CFSE: Carboxyfluorescein succinimidyl ester
CM: Conditioned medium
CP: Canonical Pathway
CXCL10: CXC-Ligand 10
CXCL11: CXC-Ligand 11
CXCL9: CXC-Ligand 9
CXCR4: C-X-C Motif Chemokine Receptor 4
EFS: Elongation factor 1a short promoter
GBP5: Guanylate Binding Protein 5
HLA-F: Major Histocompatibility Complex, Class I, F
IDO1: Indoleamine 2,3-dioxygenase
IFI27: Interferon Alpha Inducible Protein 27
IFI44L: Interferon Induced Protein 44 Like
IFN-γ: Interferon gamma
IL-10: Interleukin 10
IL-6: Interleukin 6
IL-8: Interleukin 8
IPA: Ingenuity Pathway Analysis
ISCT: International Society for Cell & Gene Therapy
MCP-1: Macrophage chemoattractant protein 1
MSC: Mesenchymal stromal cells
MSC10: Mesenchymal stromal cell medium with 10% human serum
MSC15: Mesenchymal stromal cell medium with 15% human serum
MSC-EM: Mesenchymal stromal cell explant monolayer
ns: Not significant
NOD: Non-obese diabetic
PBMCs: Peripheral blood mononuclear cells
PBS: Phosphate buffered saline
RCUG: Research Core Unit Genomics
RGB: Red green blue
RNA: Ribonucleic acid
SFFV: Spleen focus-forming virus promoter
Std: Standard
TNF-α: Tumor necrosis factor alpha
UBD: Ubiquitin D
UC: Umbilical cord
UCP: Umbilical cord piece
UR: Upstream regulator
