# Supplementary figures and images for: Umbilical cord as a long-term source of activatable mesenchymal stromal cells for immunomodulation
Source: Stem Cell Res Ther. 2019 Sep 23;10:285. doi: 10.1186/s13287-019-1376-9 (PMC6755709; doi:10.1186/s13287-019-1376-9)

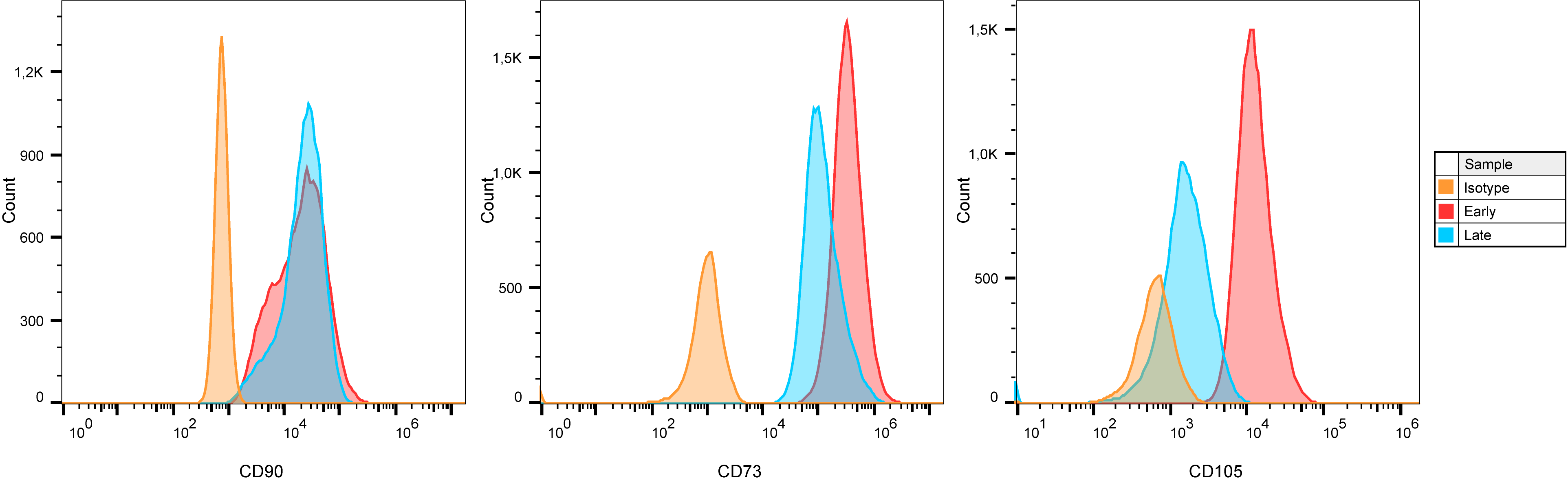

Supplement: Supplementary file 1 — Figure S1. Antibody staining of early and late induced MSC-EMs. Early and late induced MSC-EMs were stained with antibodies for CD73, CD90, and CD105 to confirm the mesenchymal stromal cell identity of the cultures. (TIF 537 kb) [file 13287_2019_1376_MOESM1_ESM.tif]
